# When to address fatigue in juvenile idiopathic arthritis during clinical visits based on the JAMAR

**DOI:** 10.1093/rheumatology/keaf325

**Published:** 2025-06-09

**Authors:** Anouk Vroegindeweij, Sabine Musterd, Sanne Nijhof, Alessandro Consolaro, Sebastiaan Vastert, Sytze De Roock, Joost Swart

**Affiliations:** Department of Social Pediatrics, Wilhelmina Children's Hospital, University Medical Center Utrecht, Utrecht University, Utrecht, The Netherlands; Department of Pediatric Rheumatology/Immunology, Wilhelmina Children’s Hospital, University Medical Center Utrecht, Utrecht University, Utrecht, The Netherlands; Department of Social Pediatrics, Wilhelmina Children's Hospital, University Medical Center Utrecht, Utrecht University, Utrecht, The Netherlands; Strategic program of Child Health, Faculty of Medicine, Utrecht University, Utrecht, The Netherlands; Department of Neurosciences, Rehabilitation, Ophthalmology, Genetics and Maternal-Infantile Sciences (DiNOGMI), University of Genoa, Genoa, Italy; Department of Pediatric Rheumatology/Immunology, Wilhelmina Children’s Hospital, University Medical Center Utrecht, Utrecht University, Utrecht, The Netherlands; Strategic program of Child Health, Faculty of Medicine, Utrecht University, Utrecht, The Netherlands; Department of Pediatric Rheumatology/Immunology, Wilhelmina Children’s Hospital, University Medical Center Utrecht, Utrecht University, Utrecht, The Netherlands; Department of Pediatric Rheumatology/Immunology, Wilhelmina Children’s Hospital, University Medical Center Utrecht, Utrecht University, Utrecht, The Netherlands; Strategic program of Child Health, Faculty of Medicine, Utrecht University, Utrecht, The Netherlands

**Keywords:** checklist individual strength-8, fatigue, juvenile arthritis multidimensional assessment report, juvenile idiopathic arthritis, quality of life

## Abstract

**Objectives:**

Fatigue is a prevalent but overlooked issue among patients with Juvenile Idiopathic Arthritis (JIA) in clinical practice. The internationally widely used Juvenile Arthritis Multidimensional Assessment Report (JAMAR) does not include items on fatigue. We evaluated whether items from its Quality of Life (QoL) section could be used as proxy measurement for fatigue, prompting practitioners to address issues with fatigue during a clinical visit.

**Methods:**

105 JIA patients at the Wilhelmina Children’s Hospital (WCH) completed the JAMAR and Checklist Individual Strength-8 (CIS-8), a questionnaire with validated cut-off score for severe fatigue in rheumatic conditions. Correlations were inspected to test whether QoL items 3 (difficulty with energy-demanding activities) and 9 (concentration or attention issues) correlated strongest with severe fatigue. With binary logistic regressions and ROC analyses, the proxy’s predictive value and cut-off were determined. The proxy was re-used in the EPOCA cohort for comparison.

**Results:**

The proposed items showed the strongest correlation with severe fatigue (*r*_item3_ = 0.644 and *r*_item9_ = 0.565). Their sum score represented the proxy (range 0–6). The proxy was a significant predictor of severe fatigue controlled for age, sex, and disease activity (*P* < 0.001). Area Under the Curve was 0.911, sensitivity 90% and specificity 69.6% with cut-off score ≥1. According to the proxy, fatigue should be addressed in 58.1% of WCH patients and in 56.6% of the EPOCA cohort.

**Conclusion:**

The proxy can be used to estimate whether issues with fatigue should be explored during clinical visits. To quantify fatigue severity levels, we recommend using the CIS-8 or another fatigue questionnaire.

Rheumatology key messages

- Many patients with JIA experience (severe) fatigue, which often goes unnoticed unless directly addressed.
- The JAMAR includes no fatigue items, but this study identifies two JAMAR-items that function as a proxy measurement of fatigue.
- Until a specific fatigue item has been added, the proxy may help practitioners identify when fatigue should be addressed.

## Introduction

Juvenile Idiopathic Arthritis (JIA) affects at least 0.2 out of every 1000 children [1]. JIA can lead to daily experiences of pain and joint stiffness or present itself with fluctuating levels of disease activity over time [1]. Regardless of the level of disease activity, 60–76% of all JIA patients report experiencing fatigue [1]. Approximately 25% even report suffering from severe fatigue [2]. Severe fatigue can make a large, negative impact on daily life, with reductions in physical and social functioning, school attendance and performance, and mental well-being [2–4]. Patients, caregivers, healthcare professionals, and researchers have tried to draw attention to fatigue as a pressing issue during both active disease and remission [1, 5–7].

There are ways to increase focus on fatigue in clinical practice. The most straightforward way is to start asking patients about their fatigue levels systematically. The Juvenile Arthritis Multidimensional Assessment Report (JAMAR) is a questionnaire widely used in clinical JIA care to monitor disease activity and quality of life (QoL) [8]. However, it does not contain specific items on fatigue. Instead, practitioners could ask patients to complete another validated questionnaire regarding fatigue, such as the Checklist Individual Strength (CIS)-8 [9], but this increases burden for all patients. Ideally, only one new item would be added to the JAMAR to identify patients with (probable) fatigue. However, as this demands a long process of item conceptualization, validation, and implementation in different countries, we considered it worth exploring whether the JAMAR already contains items that relate to fatigue—items that could serve as an indirect or proxy measurement of fatigue.

As fatigue is strongly associated with (health-related) QoL [1–5], we focused on the QoL section of the JAMAR. At face value, we expected that two items from this section would perform well together as a proxy. These two items inquire about difficulty with energy-demanding activities and concentration issues in the past 4 weeks. With this study, we explored the usefulness of these two items as a proxy for fatigue. We expected that the sum score of the two items would indicate the probability of fatigue being an issue that needs to be addressed by practitioners during the patient’s clinical visit.

## Methods

### Patients

The main part of this study focuses on 105 patients diagnosed with JIA at the Wilhelmina Children’s hospital (WCH), part of University Medical Center (UMC) Utrecht, The Netherlands, between January 2017 and October 2023. All patients were aged between 8 and 18. Their data were derived from the WCH’s PROactive cohort [10]. In the PROactive cohort, patients (and parents if patients are younger than 16) provided consent to use their data for research purposes. The PROactive cohort was classified by the institutional review board of UMC Utrecht as exempt from the Medical Research Involving Humans Act, case number 16/707-C. For a subpart of this study, we used data from EPOCA, a cohort study which includes 8618 patients from over 55 different countries [11]. All participating centres obtained approval of the EPOCA cohort by their local institutional review boards, and patients (and parents when appropriate) provided consent to use their data for research purposes [11].

### Measurements

All 105 patients completed two questionnaires at the same day or at least within 31 days of each other. The first questionnaire was the JAMAR, from which we retrieved all responses to the QoL items, as well as the active joint count (AJC) to get an indication of disease activity. The QoL items required patients to reflect on the past 4 weeks and indicate on a 4-point Likert scale whether they have experienced issues regarding this item (0 = Never, 1 = Sometimes, 2 = Often, 3 = Every day). Out of all 10 items, the two items of interest were: ‘In the past four weeks, I have had any difficulty carrying out activities that require a lot of energy such as running, playing football, dancing etc’ and ‘In the past 4 weeks, I have had any difficulty concentrating or paying attention’. These are, respectively, item numbers 3 and 9 from the QoL section. Their sum score can range from 0 to 6 (ordinal scale). We expect that sum scores of 0 indicate no need for healthcare professionals to address fatigue during a clinical visit, whereas higher sum scores do indicate a higher probability of fatigue being an issue that needs to be addressed.

The second questionnaire was the CIS-8, a reliable and validated questionnaire to measure experienced fatigue severity [9]. The questionnaire ranges from 8 to 56, with higher scores indicating higher levels of fatigue. The validated cut-off score for severe fatigue in rheumatic conditions is >34 [12].

Demographic information was retrieved from the WCH’s PROactive cohort in which all 105 patients were enrolled [10].

### Statistical analyses

Patients were classified as ‘severely fatigued’ if their CIS-8 total score was >34. We compared baseline characteristics of patients with and without severe fatigue using t-tests or *χ*^2^ tests when appropriate. Point-biserial correlations were used to test whether QoL items 3 and 9, our proposed proxy, were also the items most strongly correlated with severe fatigue. Next, we tested whether the proxy was a predictor of severe fatigue using a binary logistic regression. We added the proxy and control variables age, sex, and disease activity stepwise to the model. The dependent variable was presence or absence of severe fatigue according to the CIS-8 total score. Model fit was evaluated using *R*^2^ and -2 Log Likelihood (-2LL) values, with higher *R*^2^ and lower -2LL values indicating better model fit. Then, we performed a ROC analysis to establish a cut-off score for probable fatigue that needs to be addressed during a clinical visit. We evaluated the Area Under the Curve (AUC), sensitivity and specificity for the total sample and for subsamples based on age, sex and disease activity. We compared the proxy score of patients with and without severe fatigue using a Mann–Whitney-*U* test. Effect size *r* ($\frac{Z-\mathrm{score}}{\surd N}$) was calculated and values of >0.5 were considered large [13]. Finally, we compared the proxy response distributions between our sample and the EPOCA sample to determine whether both groups identified an equivalent number of patients with probable (severe) fatigue using the proxy. All analyses and figures were performed and created in SPSS version 29.0.2.0.

## Results

### Total sample description

Thirty out of 105 patients were identified as severely fatigued according to the CIS-8 total score (28.57%). These patients were significantly older and more often female compared with patients without severe fatigue. Patients with severe fatigue also reported lower QoL (as higher scores indicate lower quality). Details are summarized in Table 1.

**Table 1.** Patient characteristics and group comparisons with and without severe fatigue

Characteristics	Total sample (*N* = 105)	With severe fatigue (*n* = 30)	Without severe fatigue (*n* = 75)	*P* value
Age in years, mean (s.d.)	14.92 (2.50)	16.26 (1.74)	14.41 (2.21)	0.000
Female, frequency (%)	71 (67.60%)	28 (93.31%)	43 (57.33%)	0.000
Disease duration in years, mean (s.d.)	7.18 (4.08)	7.73 (4.34)	7.07 (4.09)	0.423
Disease active, frequency (%)^a^	25 (23.80%)	14 (46.70%)	11 (14.70%)	0.002
Disease in remission, frequency (%)^a^	67 (63.80%)	13 (43.30%)	54 (72.00%)	0.002
QoL total score,^b^ mean (s.d.)	4.35 (4.86)	9.17 (4.99)	2.39 (3.20)	0.000
CIS-8 total score,^c^ mean (s.d.)	25.55 (13.82)	44.23 (5.98)	18.05 (7.47)	0.000

T test or *χ*^2^ tests were used appropriately for group comparisons with *α* = 0.05.

a Disease activity data based on the AJC and was missing for 13 JIA patients (12.40%). We considered disease as active if AJC was > 0.

b JAMAR’s *QoL* section with higher total scores indicating lower *QoL*.

c Checklist Individual Strength-8 with higher total scores indicating more severe fatigue.

### JAMAR *QoL* correlations with severe fatigue

All QoL items were significantly correlated with severe fatigue (all *P* < 0.01). In line with our hypothesis, items 3 and 9 displayed the strongest correlation with severe fatigue. The correlations are presented in Table 2. Because all items are correlated with fatigue, we will also test the predictive value of the QoL total score in the next section.

**Table 2.** Point-biserial correlations with severe fatigue

QoL	Item 1	Item 2	Item 3	Item 4	Item 5	Item 6	Item 7	Item 8	Item 9	Item 10
***r***	0.291	0.517	**0.644**	0.301	0.520	0.379	0.313	0.264	**0.565**	0.407

Shown are the correlations between the JAMAR QoL items and being severely fatigued according to the CIS-8 questionnaire (i.e. CIS-8 total score >34). QoL item 1 inquires after difficulty taking care of yourself, QoL item 2 after walking, QoL item 3 after energy-demanding activities, QoL item 4 after at-school activities or playing with friends, QoL item 5 after pain, QoL item 6 after feeling sad or depressed, QoL item 7 after feeling nervous or anxious, QoL item 8 after having trouble getting along with other children, QoL item 9 after concentration or attention difficulties, and QoL item 10 after feeling dissatisfied with your physical appearance or abilities. *r* = correlation coefficient. Printed in bold are the correlations of the proxy items.

### Proxy as predictor of severe fatigue

The first binary logistic regression model included the control variables age, sex, and disease activity only. This model had an *R*^2^ of 0.367 and -2LL of 83.785. Age and sex were significant predictors of being severely fatigued according to the CIS-8 total score. Adding the proxy in the second regression model improved the fit of the model, indicated by a *R*^2^ increase to 0.727 and -2LL decrease to 45.712. In this model, only the proxy was a significant predictor of being severely fatigued (see Table 3). Thus, controlled for age, sex, and disease activity, an increase of 1 unit on the proxy (score range: 0–6) increases the odds ratio of having to address fatigue during a clinical visit with 4.656 units. Replacing the proxy with the QoL total score decreased model fit (*R*^2^ = 0.598, -2LL = 61.302), indicating that the proxy is a better predictor of severe fatigue.

**Table 3.** Output of the second binary logistic regression model

Predictors	*B*	S.E.	*Wald*	df	*Exp*(*B*)	*P*
Age	0.340	0.193	3.100	1	1.404	0.078
Sex (female)	−1.767	1.007	3.077	1	0.171	0.079
AJC	0.110	0.214	0.265	1	1.116	0.607
**Proxy**	**1.538**	**0.345**	**19.899**	**1**	**4.656**	**<0.001**
Constant	−8.198	3.058	7.188	1	0.000	0.007

*α*  =  0.05. s.e.: standard estimate; AJC: active joint count, representing disease activity. Exp(B) = Odds ratio. Dependent outcome: severe fatigue (i.e. CIS-8 total score >34). Printed in bold are the outcomes related to the proxy.

### Comparison of proxy scores between patients with and without severe fatigue

Patients with severe fatigue responded significantly higher on the proxy (*M *= 3.07, s.d.* = *1.34) compared with those without severe fatigue (*M = *0.65, s.d.* = *0.89). The Mann–Whitney *U* test yielded a test statistic of 199.50, *Z*-score of 6.879, and a large effect size of *r *= 0.67 (*P* < 0.001).

### Proxy cut-off score: when to address probable fatigue during clinical visit

**Figure 1.**
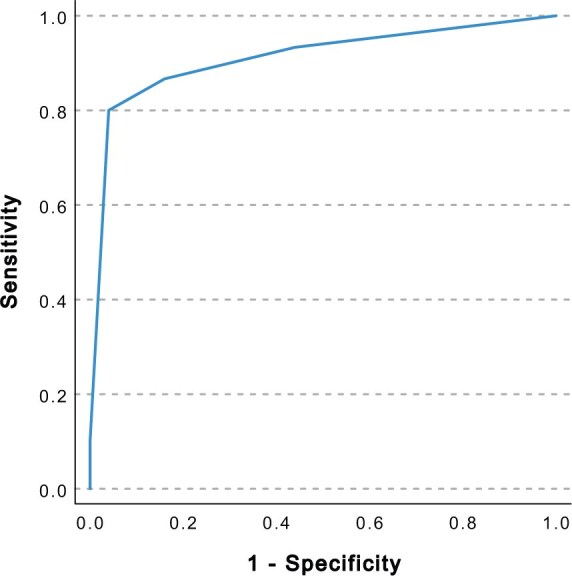
ROC curve of the fatigue proxy

The ROC analysis yielded an AUC of 0.911 in the total sample (CI_95%_ = 0.838–0.984). Fig. 1 presents the ROC curve of the proxy. The coordinates of the ROC curve are displayed in Table 4. With proxy cut-off score ≥1, sensitivity was 90.0% and specificity was 69.6%. This means that the probability of skipping a discussion about fatigue when it should be addressed is 10% (reflecting the false negatives), whereas the probability of discussing fatigue when it is not an issue is 30.4% (reflecting the false positives).

**Table 4.** Coordinates of the fatigue proxy’s ROC curve

Proxy score ≥	Sensitivity	Specificity
0	0.967	0.277
**1**	**0.900**	**0.696**
2	0.834	0.899
3	0.567	0.973
4	0.217	0.993
5	0.067	1.000
6	0.017	1.000

The present sample consisted of 30 patients with severe fatigue and 75 without severe fatigue. Having severe fatigue was defined by a CIS-8 total score of >34. Printed in bold are the sensitivity and specificity values of the proposed cut-off score.

### Equivalent percentage in the WCH sample and EPOCA cohort

With ≥1 as proxy cut-off, fatigue should be addressed as probable issue in 61 out of 105 WCH patients (58.1%). In the EPOCA cohort, an equivalent percentage of 56.6% was identified.

## Discussion

The JAMAR does not include specific items on fatigue, despite it being a severe issue in many patients with JIA [1, 5–7]. Therefore, we investigated the use of two JAMAR *QoL* items as proxy measurement of fatigue. These two items inquire after difficulties carrying out energy-demanding activities and concentration or attention issues in the past four weeks. In line with our expectation, we found that this proxy measurement can be a useful tool in clinical practice. Proxy scores of ≥1 indicate that fatigue should be addressed as potential issue during a patient’s clinical visit. In other words, if patients respond sometimes, often, or every day having issues concerning at least one of the two items, practitioners are encouraged to prompt a conversation about fatigue.

Point-biserial correlation tests showed that out of all ten QoL items, our proposed proxy items (items 3 and 9), correlated most strongly with being severely fatigued according to the validated CIS-8 questionnaire. Binary logistic regressions added that the proxy was a significant predictor of severe fatigue when controlled for age, sex, and disease activity represented by the AJC. Patients with severe fatigue scored significantly higher on the proxy than patients without severe fatigue. Furthermore, ROC analysis yielded an excellent AUC of 0.911 for the proxy. Using ≥1 as cut-off (on a scale of 0–6), fatigue should be addressed as potential issue in an equivalent number of patients in our sample (*N* = 105) and the much larger EPOCA cohort (*N* = 8618), namely, respectively, 58.1% and 56.6%, which shows preliminary support for the generalized use of the proxy across countries.

Some practitioners may prefer ≥2 as cut-off score for the proxy. With this score, sensitivity would be 83.4% and specificity 89.9%. This is a large improvement in specificity, which lowers the number of times practitioners must ask whether fatigue is an issue when in fact it is not. Yet, the higher cut-off increases the number of times that practitioners falsely skip the discussion by 7.6%. We preferred the cut-off with highest sensitivity, as we considered the effort of asking about fatigue during a clinical visit as small.

Importantly, the proxy should only be used to indicate whether fatigue should be discussed during a clinical visit. With cut-off score ≥1, the sensitivity of the proxy is excellent (90%), but the specificity (69.6%) limits the use of the proxy for other purposes. For example, the proxy cannot be used to determine whether a patient suffers from severe fatigue. If practitioners (or researchers) want to quantify the level of fatigue as a next step, we recommend using validated questionnaires such as the CIS-8 or its shorter version named the paediatric Short Fatigue Questionnaire [9, 14], the Paediatric Quality of Life Inventory Multidimensional Fatigue Scale (PedsQL-MFS) [15, 16], or others (for more examples, see ref. 12). If patients are classified with severe fatigue issues according to validated questionnaires and ask for help, the final step would be to direct these patients to fatigue management programs or intervention studies available locally or online (see e.g. Refs. [17–22]).

Ultimately, we encourage the development and addition of a single item directly measuring fatigue to the JAMAR. Until then, the proxy may be used by practitioners as a conversation starter during clinical visits.

## Data Availability

WCH data are available upon reasonable request, please contact J.F.S. (j.f.swart@umcutrecht.nl) and S.L.N. (s.l.nijhof@umcutrecht.nl).
